# Novel action of apolipoprotein E (ApoE): ApoE isoform specifically inhibits lipid-particle-mediated cholesterol release from neurons

**DOI:** 10.1186/1750-1326-2-9

**Published:** 2007-05-15

**Authors:** Jian-Sheng Gong, Shin-ya Morita, Mariko Kobayashi, Tetsurou Handa, Shinobu C Fujita, Katsuhiko Yanagisawa, Makoto Michikawa

**Affiliations:** 1 Department of Alzheimer's Disease Research, National Institute for Longevity Sciences, 36-3, Morioka, Obu, Aichi 474-8522, Japan; 2 Pharmaceuticals and Medical Devices Agency, Tokyo, Japan; 3 Department of Biosurface Chemistry, Graduate School of Pharmaceutical Sciences, Kyoto University, Kyoto 606-8501, Japan; 4 Mitsubishikagaku Institute of Life Sciences, 11 Minamiooya, Machida, Tokyo 194-8511, Japan

## Abstract

**Background:**

Since the majority of apolipoprotein E (apoE) existing in the cerebrospinal fluid is associated with high-density lipoprotein (HDL), one should focus on the role of the apoE-HDL complex rather than on that of free apoE in cholesterol metabolism in the central nervous system. However, the apoE-isoform-specific effect of apoE-HDL on cholesterol transport remains unclarified.

**Results:**

Here we show that apoE3-HDL induced a marked cholesterol release from neurons, while apoE4-HDL induced little. To elucidate the mechanism underlying this phenomenon, we used a complex of lipid emulsion (EM) with recombinant apoE3 or apoE4 (apoE-EM) at various apoE concentrations. When a small number of apoE molecules were associated with EM, apoE3- and apoE4-EM, induced a marked cholesterol release to a level similar to that induced by EM alone. However, when apoE at given concentrations was incubated with EM, apoE3-EM induced a marked cholesterol release, while apoE4-EM induced little. Under these conditions, a greater number of apoE4 molecules were associated with EM than apoE3 molecules. When an increasing number of apoE molecules were associated with EM, both apoE3-EM and apoE4-EM induced little cholesterol release. Preincubation with β-mercaptoethanol increased the number of apoE3 molecules associated with EM similar to that of apoE4 molecules, indicating that the presence (apoE3) or absence (apoE4) of intermolecular disulfide bond formation is responsible for the association of a greater number of apoE4 molecules to EM than apoE3 molecules.

**Conclusion:**

These results suggest that although apoE and a lipid particle are lipid acceptors, when apoE and a lipid particle form a complex, apoE on the particle surface inhibits the lipid particle-mediated cholesterol release from cells in an apoE-concentration-dependent manner.

## Background

It has been shown that the prevalence of Alzheimer's disease (AD) is associated with the polymorphisms of genes related to cholesterol metabolism, including *apolipoprotein E (apoE)*[1], *ATP-binding cassette transporter A1 (ABCA1) *[2], and *CYP46*, the gene encoding cholesterol 24-hydroxylase [3,4]. However, before discussing the association of altered cholesterol metabolism with AD pathogenesis, one should delineate mutual interaction between cholesterol metabolism in the circulation and that in the central nervous system across the blood-brain barrier, and also determine how cholesterol is transported within the central nervous system and how altered cholesterol metabolism induces AD pathologies. In the central nervous system, apoE is one of the major lipid acceptors [5,6] and interacts with ABCA1 [7] to remove cholesterol from cells and generate HDL particles [8] in an apoE-isoform-specific manner [9-11]. This isoform-specific action of free apoE to remove cholesterol and to generate HDL would be a possible cause for the altered cholesterol metabolism in an AD brain. On the other hand, it was shown that the majority of apoE existing in cerebrospinal fluid (CSF) and culture media is associated with HDL and the free form of apoE is at a very low level in the CSF [5,6] and culture media [10,12]. Thus, to determine the apoE-isoform-specific cholesterol transport in the central nervous system, one should focus on the role of the apoE-HDL complex rather than on that of free apoE.

Many studies have shown that HDL stimulates cholesterol release from cultured cells [13-15]. It is believed that this removal of cellular cholesterol induced by HDL involves at least two different mechanisms working cooperatively. One involves the biochemical pathway mediated by apolipoproteins [16,17]. The other involves the physicochemical pathway for the bidirectional movement of cholesterol mediated by aqueous diffusion mechanism [18,19]. However, how these two acceptors contribute and modulate the cholesterol release remains to be clarified. Our recent finding that the apoE-isoform-specific ability to generate HDL is associated with an apoE-isoform-specific ratio of apoE molecules per HDL particle [10] led us to examine the effect of apoE3- and apoE4-containing HDLs or lipid emulsions (EMs) at different apoE ratios on cholesterol release from neurons. Here we show that apoE3-HDL induces a strong cholesterol release, while apoE4-HDL induces a very weak release, and that this isoform-specific effect of apoE associated with lipid particle (HDL or EM) is due to the finding that (1) apoE4 has a higher affinity to lipid particles and thus a greater number of apoE4 molecules bind to lipid particles than apoE3, and (2) with increasing number of apoE molecules covering the surface of lipid particle, both apoE3 and apoE4 inhibit the lipid-particle-mediated cholesterol release. These results suggest that both apoE and a lipid particle are strong lipid acceptors; however, when apoE forms a complex with a lipid particle, apoE on the particle surface inhibits the lipid-particle-mediated cholesterol release by covering its surface.

## Results

### ApoE-isoform-specific lipid release mediated by apoE3- and apoE4-containing HDL

**Figure 1:**
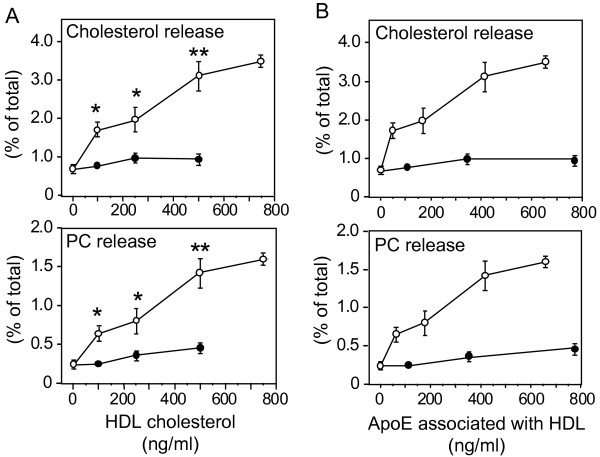
**Characterization of cholesterol and PC release from cultured neurons in the presence of apoE3-HDL or apoE4-HDL**. Neurons labeled with 37 Bq/ml [^14^C] acetate were cultured for 3 days at 37°C. These neurons were washed in DMEM three times and incubated in DMEM for 1 h in the presence of apoE3-HDL or apoE4-HDL at various ApoE or HDL cholesterol concentrations, and the amounts of released [^14^C]-labeled cholesterol and PC were determined. **A) **The amounts of cholesterol and PC released, as induced by apoE3-HDL (○), were significantly greater than those of cholesterol and PC released, as induced by apoE4-HDL (●). **B) **The amounts of cholesterol and PC are plotted against the concentrations of apoE associated with HDL. Data are means ± S.E. of four samples. *p < 0.05 and **p < 0.001 vs apoE4-HDL at various cholesterol concentrations. Six independent experiments show similar results.

**Figure 2:**
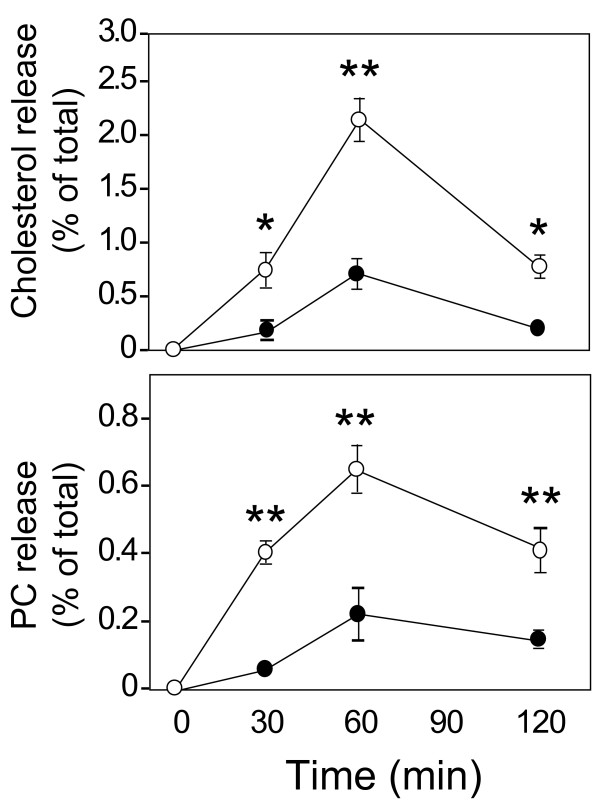
**Time-dependent release of cholesterol and PC from cultured neurons in the presence of apoE3-HDL or apoE4-HDL**. The kinetics of cholesterol and PC release in the presence of apoE3- or apoE4-HDL at a cholesterol concentration of 500 ng/ml were determined. Both apoE3- (○) and apoE4-HDL (●) induced cholesterol and PC release in a time-dependent manner. Data are means ± S.E. of four samples. *p < 0.001 and **p < 0.0001 vs apoE4-HDL. Six independent experiments showed similar results.

Human apoE3- and apoE4-containing HDL (apoE3-HDL and apoE4-HDL, respectively) were obtained from the conditioned media of each culture as described in the "Experimental Procedures". As many previous studies demonstrated, apoE3-HDL promoted cholesterol and phosphatidylcholine (PC) release from neurons in an HDL-dose-dependent manner (Fig. 1A). In contrast, surprisingly, the amounts of cholesterol and PC released from cultured neurons in the presence of apoE4-HDL remained very low at any HDL-cholesterol concentrations examined (Fig. 1A). Because our previous study demonstrated that apoE4-HDL contains apoE molecules twofold those in apoE3-HDL per particle [10], we determined the amount of apoE molecules in each HDL fraction added and plotted against the amount of cholesterol and PC released at various apoE concentrations. As shown in Fig. 1B, even when a comparable or a greater amount of apoE molecules was included in the apoE4-HDL than that in the apoE3-HDL, it did not promote lipid release, either (Fig. 1B). Analysis of the time dependence of lipid release mediated by apoE-HDL showed that lipid release mediated by apoE-HDL reached the peak 60 min following the addition of apoE3-HDL and apoE4-HDL (Fig. 2).

### Effect of apoE-emulsion complex on cholesterol release from cultured neurons

**Figure 3:**
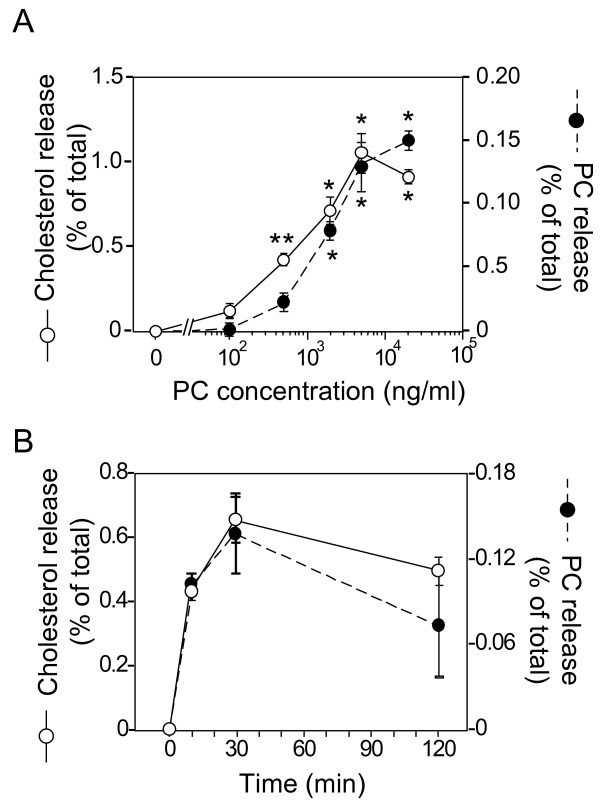
**EM mediated cholesterol and PC release from cultured neurons**. Neurons labeled with 37 Bq/ml [^14^C]acetate were cultured for 3 days at 37°C. These neurons were washed in DMEM three times and further incubated in DMEM for 1 h **(a) **or various times **(b) **in the presence of EM at various PC concentrations. The conditioned media were then collected and the amounts of [^14^C]-labeled cholesterol and PC were determined. **(a) **EM-induced cholesterol (○) and PC (●) release from cultured neurons occurs in an EM-PC-dose-dependent manner. **(b) **The time-dependent release of cholesterol (○) and PC (●) from cultured neurons in the presence of EM at PC concentration of 5 μg/ml is shown. Data are means ± S.E. of four samples. *p < 0.0001 and **p < 0.001 vs PC at 0 ng/ml and time at 0 h. Four independent experiments showed similar results.

To elucidate the mechanism underlying the apoE-isoform-specific effect on cholesterol release mediated by apoE-HDL, we used a complex consisting of lipid emulsion (EM) and recombinant human apoE3 or apoE4, because one cannot modulate the number of apoE molecules associated with HDL, but one can modulate apoE number associated with EM. Using this system, we can investigate the effect of apoE on EM-mediated lipid efflux. EM is generated using phosphatidylcholine and triolein and its diameter was determined as described in the Experimental Procedures, and the apoE-EM complexes were then re-isolated before use for the various assays performed. When EM was added to the neuronal cultures in which cholesterol was labeled with ^14^C-acetate, cholesterol and PC were released in a time- and an EM-dose-dependent manner (Fig. 3), showing that EM serves as a lipid acceptor to release cholesterol. Time-dependent kinetics in terms of lipid release showed that the level of lipids released into the conditioned media saturated 30 min following the commencement of treatment. We determined the amounts of cholesterol and PC released using EM particles at a PC concentration of 5 μg/ml 60 min following the commencement of treatment in the following experiments performed.

### ApoE-dose-dependent inhibition of EM-mediated lipid release

**Figure 4:**
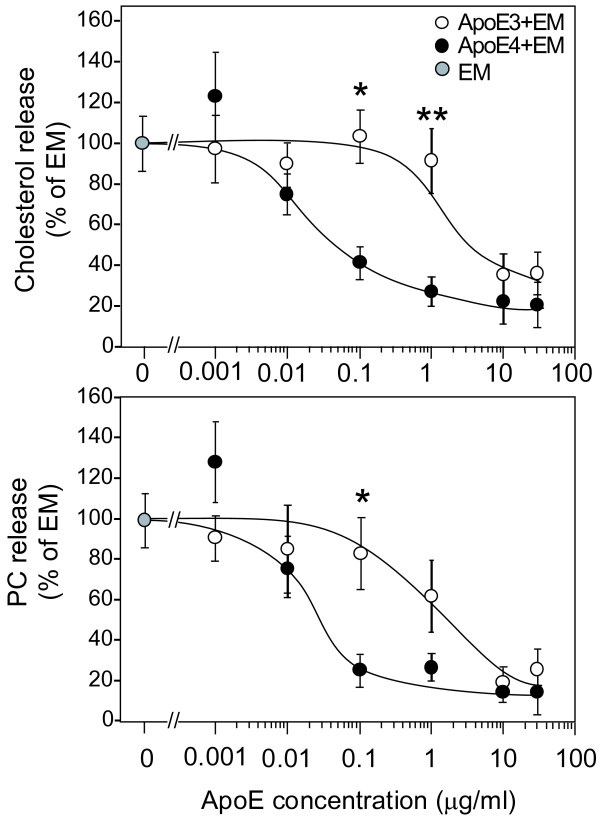
**Effect of apoE associated with EM on EM-mediated cholesterol and PC release**. Cultured neurons were incubated with serum-free N2 medium containing 37 Bq/ml [^14^C]acetate for 3 days at 37°C. These neurons were washed in DMEM three times and further incubated in DMEM for 1 h in the presence of the apoE-EM complex at various apoE concentrations. The conditioned media were then collected and the amounts of [^14^C]-labeled cholesterol and PC released were determined. The amounts of cholesterol and PC released into the conditioned media in the presence of apoE3-EM (○) or apoE4-EM (●) are shown. Data are means ± S.E. of four samples. *p < 0.01 and **p < 0.005 vs apoE4-EM. Six independent experiments showed similar results.

We examined the dose-dependent effect of apoE associated with EM on apoE-EM-mediated lipid release. EM alone and the apoE3- and apoE4-EM complexes induced lipid release from neurons; however, with increasing concentration of apoE incubated with EM, the levels of lipid released induced by both apoE3- and apoE4-EM complexes decreased (Fig. 4). However, when the apoE-EM complexes were generated by the incubation of apoE at concentrations of 0.1 and 1 μg/ml with EM (at a PC concentration of 50 μg/ml) and were applied at a PC concentration of 5 μg/ml, apoE3-EM strongly induced cholesterol release, whereas apoE4-EM induced little. The weight ratio of PC per apoE calculated in each treatment is shown in Table 1. The ratio of ratio of PC per apoE at apoE concentrations of 0.1,1,10, and 30 μg/ml was greater in the apoE3-EM complex than in the apoE4-EM complex, indicating that apoE4 has higher binding affinity to EM than apoE3. Interestingly, when the ratios (PC/apoE) were (a) 496 ± 97 (for apoE3) and 269 ± 85 (for apoE4), and (b) 23 ± 6 (for apoE3) and 16 ± 6 (for apoE4), which were obtained by incubating EM with apoE at concentrations of (a) 0.1 and (b) 1.0 μg/ml, respectively (Table 1), the apoE-isoform-specific cholesterol release was observed (Fig. 4) as was the case for apoE-HDL (Fig. 1), that is, apoE3-EM and apoE3-HDL induced cholesterol release from neurons, whereas apoE4-EM and apoE4-HDL induced little release.

**Table 1:** Binding of apoE3 and apoE4 molecules to emulsion particles

apoE concentration (μg/ml)		0.1	1	10	30
Weight ratio of PC per apoE	apoE3	496 ± 97	23 ± 6	7 ± 2	8 ± 3
	apoE4	269 ± 85	16 ± 6	6 ± 1	7 ± 1

Lipid emulsions were prepared by the method described previously [36] using a high-pressure emulsifier. The emulsion used had a particle size of 34.7 ± 5.2 nm and a weight ratio of TO/PC was 1.63 ± 0.07. For the generation of apoE-EM complex, apoE at various concentrations, from 0.1 to 30 μg/ml, was incubated with EM at PC concentration of 5.0 μg/ml in a 5-ml solution for 1 hr at room temperature. The apoE-EM complex was isolated and assayed by the untracentrifugation method as previously reported [24, 37]. The weight ratio of apoE and PC were determined as described in the Experimental Procedures. Data are means ± S.E. of four samples.

**Figure 5:**
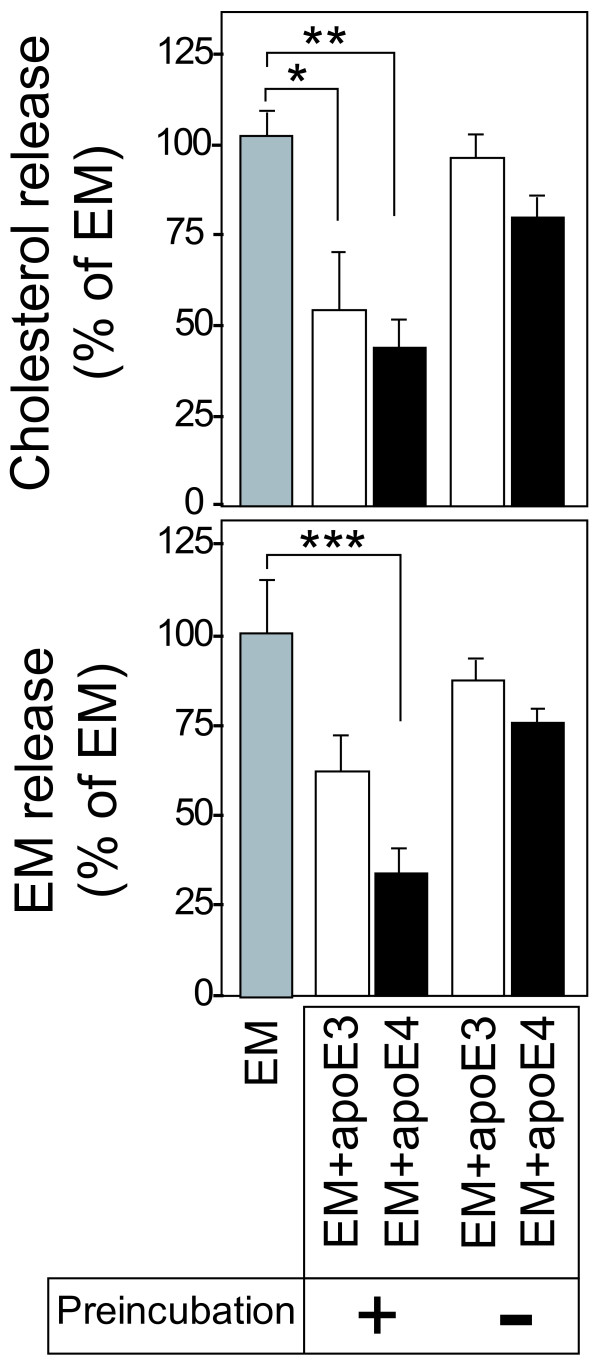
**ApoE inhibits EM-mediated lipid release when apoE is associated with EM particles**. ApoE-EM complexes were prepared as described in the Experimental Procedures. Neurons were prepared as described in Figure 4 until treatment. Neurons were treated with EM (5 μg/ml); the apoE3-EM complex (10 μg/ml, and 5 μg/ml, respectively) and the apoE4-EM complex (10 μg/ml, and 5 μg/ml, respectively), which was preincubated and isolated by centrifugation; apoE3 (10 μg/ml) and EM (5 μg/ml) or apoE4 (10 μg/ml) and EM (5 μg/ml), which were added separately. Data are means ± S.E. of four samples. *p < 0.05, **p < 0.002, and ***p < 0.02 between the values indicated. Three independent experiments showed similar results.

We next examined whether the formation of the apoE-EM complex is required for apoE to attenuate the EM-mediated lipid release. When apoE3 and apoE4 at 10 μg/ml were preincubated with 5 μg/ml EM, lipid release in the presence of apoE3-EM or apoE4-EM was significantly reduced compared with that in the presence of EM. On the other hand when they were added into the culture medium without preincubation, they did not inhibit lipid release mediated by EM (Fig. 5).

### Dimerization of apoE3 via disulfide bonds alters binding affinity of apoE3 to be similar to that of apoE4

**Figure 6:**
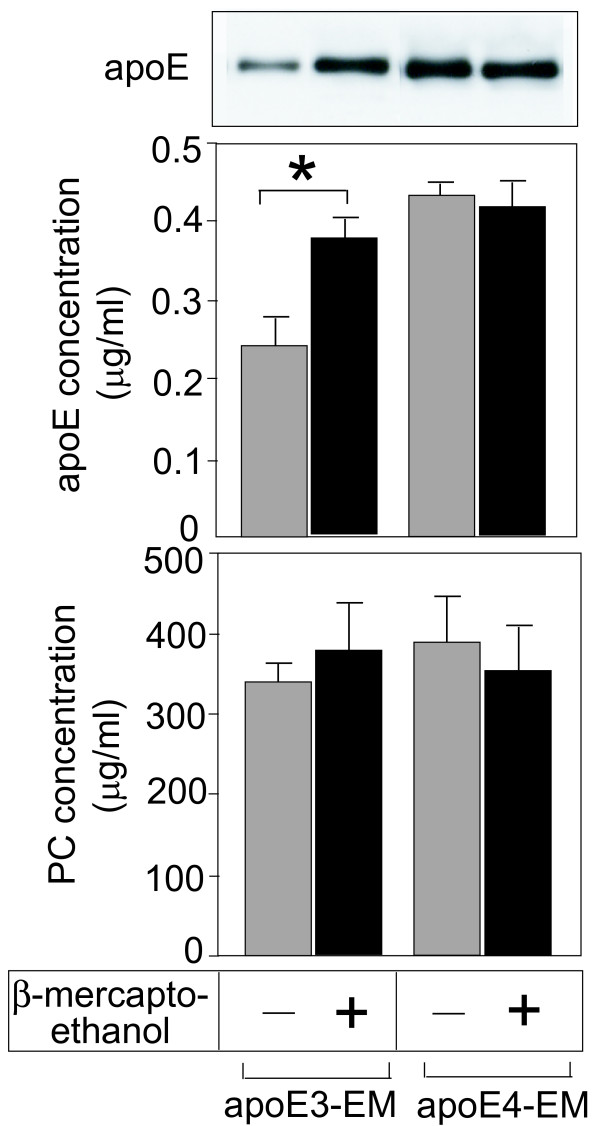
**Dimerization of apoE3 by disulfide bonds causes the difference in apoE isoform-specific binding affinity**. EM and apoE3 (or apoE4) were incubated in the presence or absence of 5% β-mercaptoethanol for 1 h and EM-PC concentrations and the number of apoE molecules in the apoE-EM complex was determined as described in the Experimental Procedures. Data are means ± S.E. of three samples. *p < 0.001. Three independent experiments showed similar results.

Because apoE3 differs from apoE4 by one amino acid at residue 152 having cysteine instead of arginine, we examined whether the dimerization of apoE3 molecules via disulfide bonds is responsible for the apoE-isoform-specific binding affinity to lipids. In the presence of 5% β-mercaptoethanol, the binding affinity of apoE3 increased to a level similar to that of apoE4 (Fig. 6). The next question is whether EM associated with apoE3 preincubated with β-mercaptoethanol, loses its ability to release cholesterol. However, this experiment was difficult to perform due to the toxic effect of β-mercaptoethanol on neurons.

## Discussion

Previous studies showed that HDL induces cholesterol release from various cell types [15,18,20]. The present study also showed that apoE3-HDL induced a strong cholesterol release, whereas apoE4-HDL induced a weak cholesterol release from neurons. As a mechanism underlying this apoE-isoform specificity, we showed a novel action of apoE, that is, although apoE is a lipid acceptor, when apoE is associated with lipid particles such as HDL and EM, apoE inhibits lipid-particle-mediated cholesterol release in an apoE-dose-dependent manner. We also found that more apoE4 molecules are associated with HDL or EM than apoE3. This may explain why apoE4 associated with HDL or EM inhibits HDL- or EM-mediated cholesterol release, whereas apoE3 does not. We also found that the dimerization of apoE3 by disulfide bonds causes the apoE-isoform dependence of the apoE binding affinity to lipids.

Cellular cholesterol release is mediated by two distinct mechanisms. One is the reaction of lipid-free apolipoproteins, such as apoE or apoAI, and any other molecules containing a certain amount of amphipathic α-helix with a cellular surface protein, ABCA1, resulting in the removal of cellular lipids and the subsequent generation of HDL-like particles [16,21]. The other one is a nonspecific physicochemical interaction causing cholesterol diffusion and its exchange between plasma membrane and lipid particles mediated by EM and liposomes. The cellular cholesterol release mediated by HDL likely involves both mechanisms, because HDL in the central nervous system contains at least two potential acceptors, apoE and lipids. One may reasonably raise questions as to which apoE isoform and to what extent lipids contribute to the HDL-mediated cholesterol release. Previous studies showed that apolipoprotein AI (apoAI) dissociated from HDL cooperatively induces cholesterol release [21,22] and that cholesterol release mediated by HDL is, in part, mediated by apoAI dissociated from HDL in an ABCA1-dependent manner [23]. However, our previous study showed that the level of free apoE in conditioned media and physiological fluid is underdetectable and almost all of the apoE is recovered from the HDL fraction [10].

**Figure 7:**
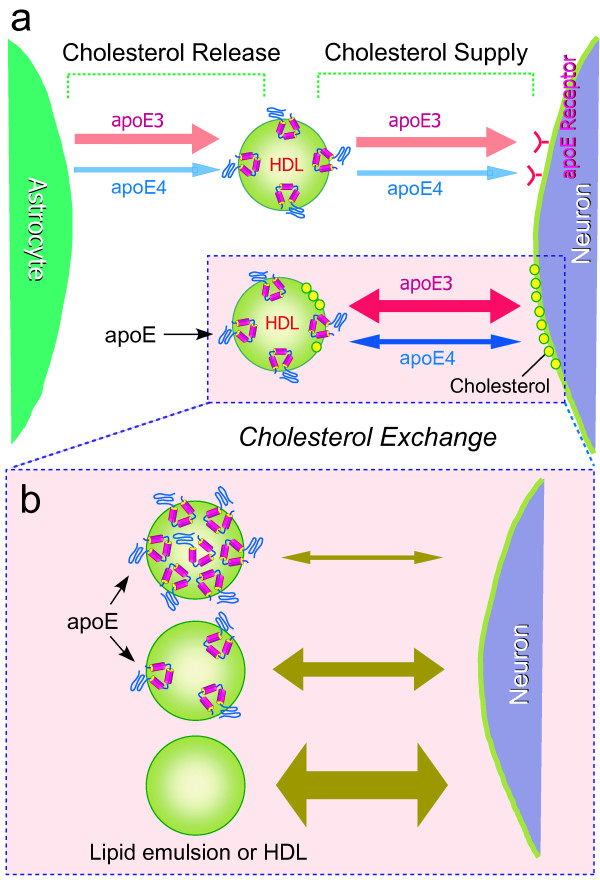
**Schema showing the apoE-isoform-specific effect of apoE-HDL and apoE-EM complex on HDL- and EM-mediated cholesterol release**. Lipid-free apoE3 released from astrocytes generates a greater number of HDL particles than apoE4 with a similar number of apoE molecules [10], indicating that apoE3-expressing astrocytes can supply more cholesterol as HDL to neurons via apoE receptors **(a)**. Lipid particles such HDL and EM have another role in cholesterol metabolism, that is, physicochemical and nonspecific cholesterol exchange between lipid particles and the cell membrane. Our present results show that with increasing number of apoE molecules on the particle surface, apoE inhibits the particle-mediated cholesterol exchange **(b)**. Because a greater number of apoE4 molecules bind to lipid particles (HDL and EM) than apoE3 molecules, apoE4 inhibits cholesterol exchange (release) by covering the lipid surface, whereas a smaller number of apoE3 molecules on lipid particle surface allows apoE3-HDL- or apoE3-EM-mediated cholesterol release **(b)**.

Moreover, as we previously reported [10], apoE4-HDL contains number of apoE molecules per particle twofold that of apoE3; however, apoE4-HDL induced a very-weak cholesterol release, whereas apoE3-HDL induced a strong cholesterol release (Figs. 1A and 1B). These results suggest that HDL harboring apoE4 on its surface does not induce cholesterol release. That is, apoE4 on the HDL surface inhibits cholesterol release mediated by HDL. This notion is confirmed by our experiments performed to determine the effect of apoE on EM-induced cholesterol release using the apoE-EM complex at various apoE concentrations on the EM surface, that is, with increasing number of apoE molecules associated with EM, not only apoE4-EM, but also apoE3-EM lose their ability to induce cholesterol release, suggesting that apoE associated with EM strongly inhibits EM-mediated cholesterol release. Interestingly, the apoE-EM complex at certain apoE concentrations and apoE/EM particle ratios induces cholesterol release in an apoE-isoform-specific manner; apoE-EM3 induces cholesterol release, but apoE4-EM does not (Fig. 4), as observed in the cases of apoE3-HDL and apoE4-HDL (Fig. 1). As shown previously [24], our data confirm that more apoE4 molecules are associated with HDL or EM than apoE3 (Table 1). These results lead us to propose a novel hypothesis that apoE molecules covering the surface of lipid particles inhibit the physicochemical interaction occurring between lipids and cell membrane. There are greater numbers of apoE4 molecules associated with EM and HDL, which in turn inhibit the EM- and HDL-mediated physicochemical exchange of cholesterol, whereas in the case of apoE3, the smaller number of apoE3 molecules on the EM or HDL surface allows cholesterol influx to EM or HDL surface from the cell membrane (Fig. 7b).

One may raise the issue that apoE-HDL/EM internalization via several apoE receptors expressed on neurons and recycled-apoE-mediated cholesterol efflux may explain apoE-isoform-dependent cholesterol efflux induced by apoE-HDL/EM. The recycle of apoE4 has been shown to be significantly decreased, resulting in a decreased level of cholesterol efflux [25]. Thus, it is possible that the poor recycling of internalized apoE4 is responsible for the reduced ability of apoE4-containing particles to release cholesterol from neurons. However, the observation that a greater amount of apoE3 associated with EM induces a smaller amount of cholesterol efflux compared with EM without apoE3 (Fig. 4) indicates that this is not the case.

It has been shown that HDL in the central nervous system contains apoE and that neurons express apoE receptors [26]. Thus, different from HDL in systemic circulation, HDL in the central nervous system is internalized into cells via apoE receptors to supply cholesterol to neurons (Fig. 7a). One may question, which is the net cholesterol transport, release from or supply to cells, in the presence of apoE-HDL and apoE-EM? It has been shown that HDL serves as the net cholesterol supplier to neurons. Previous studies showed that HDL promotes synaptogenesis [27], synaptic plasticity [28], and elongation of axons [29], and strongly suppresses cholesterol synthesis (unpublished data), indicating that HDL-cholesterol is taken up by neurons and used for axonal elongation and synaptogenesis.

If HDL, as a whole, functions as a net cholesterol supplier to neurons, what is the biological significance of apoE-HDL-mediated cholesterol release from neurons? Because physicochemical interaction causes a bidirectional cholesterol exchange (nonspecific cholesterol diffusion) between HDL cholesterol and cholesterol in the plasma membrane, it is reasonable to assume that cholesterol exchange may contribute to the maintenance of a fresh supply of cholesterol in the plasma membrane by replacing accumulated oxidized cholesterol in the membrane. Thus, the isoform-specific apoE-HDL action on cholesterol exchange suggests that the apoE3-HDL complex has a greater ability to maintain a fresh supply of cholesterol in the plasma membrane (Fig. 7b). The lower ability of apoE4-HDL may result in the accumulation of oxysterols in the plasma membrane, leading to altered membrane functions e.g., signal transduction, enzyme activities, and ion channel properties.

The last issue to be addressed is the cause of the apoE-isoform dependence of the preferential association of apoE with HDL and EM particles. Previous studies showed that apoE3 forms a disulfide-linked homodimer in plasma [30] and in culture media [10]. Thus, we determined whether this dimerization of apoE3 is responsible for the lesser number of apoE3 molecules associated with EM. The treatment of apoE3 with β-mercaptoethanol significantly increased the number of apoE3 molecules associated with EM (Fig. 6), indicating that when apoE3 remains as a monomer, more apoE3 monomers can associate with EM than apoE3 dimers. The next question is whether EM associated with apoE3, which is treated with β-mercaptoethanol, loses its ability to induce strong cholesterol release. However, this experiment is difficult to perform due to toxic effect of β-mercaptoethanol on neurons. Regarding the effect of apoE polymorphism on lipid interaction, it has been suggested that domain interaction mediated by a salt bridge between Arg-61 in the N-terminus and Glu-255 in the C-terminus, leading to a compact structure, results in the preferential binding of apoE4 to very low density lipoproteins [11,31,32]. The apoE4 domain interaction has been observed in vivo in Arg-61 knock-in mice [33]. Taken all together, it is possible that in addition to structural differences within an apoE molecule such as domain interaction, dimerization also has an effect on the apoE structure (which may also affect domain interaction), leading to the enhancement of the apoE-isoform-specific effect on the interaction between apoE and lipids.

## Conclusion

Here we have shown that although apoE and a lipid particle such as EM are lipid acceptors, when apoE and a lipid particle form a complex, apoE inhibits the lipid particle-mediated cholesterol release from cells in an apoE-dose-dependent manner, probably owing to the occupation of the surface of the lipid particle, thereby inhibiting lipid diffusion and exchange. The observation that a greater number of apoE4 molecules are associated with EM than apoE3 may explain the apoE-isoform-dependent lipid release induced by the apoE-lipid complex. Because lipid particles such HDL and EM induce physicochemical and nonspecific cholesterol exchange between lipid particles and the cell membrane, the lower ability of the apoE4-lipid complex to release lipids may result in the lower lipid replacement and accumulation of oxysterols in the plasma membrane, leading to altered membrane functions, e.g., signal transduction, enzyme activities, and ion channel properties.

## Methods

### Animals

The animal care and the experiments using animals were carried out in accordance with institutional guidelines. Mice expressing human apoE4 in place of mouse apoE were generated by the gene-targeting technique taking advantage of homologous recombination in embryonic stem cells (knock-in) as previously described [34]. ApoE3 knock-in mice were generated in the same manner except that the transgene carried apoE3 cDNA in place of apoE4 cDNA. Postnatal day 2 mice that possess the homozygous epsilon 3 (3/3) or epsilon 4 (4/4) allele, and correctly expressing human apoE3 or apoE4 proteins, respectively, were used in this study.

### Cell culture

Highly astrocyte-rich cultures were prepared according to a previously described method [10]. In brief, the brains of postnatal day 2 mice were removed under anesthesia. The cerebral cortical fragments were incubated in 0.25% trypsin and 20 mg/ml DNase I in phosphate-buffered saline (PBS) (8.1 mM Na_2_HPO_4_,1.5 mM KH_2_PO_4_O, 137 mM NaCl and 2.7 mM KC1, pH 7.4) at 37°C for 20 min. The fragments were then dissociated into single cells by pipetting. The dissociated cells were seeded in 75-cm^2 ^dishes at a cell density of 1 × 10^7 ^in Dalbecco's modified essential medium (DMEM) containing 10% FBS. After 10 days of incubation in vitro, astrocytes in the monolayer were trypsinized (0.1%) and reseeded onto six-well dishes and maintained in DMEM containing 10% FBS until use.

Neuron-rich cultures were prepared from rat cerebral cortices as previously described [35]. Dissociated cells were suspended in the feeding medium and plated onto poly-D-lysine-coated twelve-well plates at a cell density of 2 × 10^5^/cm^2^. The feeding medium consisted of DMEM nutrient mixture (DMEM/F12; 50%: 50%) and N2 supplements. More than 99% of the cultured cells were identified as neurons by immunocytochemical analysis using a monoclonal antibody against microtubule-associated protein 2, a neuron-specific marker, on day 3 of culture.

### Preparation of HDL released into conditioned media of astrocytes expressing apoE3 or apoE4

Astrocytes in 75-cm^2 ^dishes were washed in DMEM three times and incubated in 12 ml of DMEM for 5 days at 37°C. After incubation, the astrocyte culture medium was collected, centrifuged at 1, 600 × g for 15 min in a 50-ml plastic tube to exclude cell debris, and adjusted to a discontinuous sucrose gradient, which was prepared in a 14 × 89 mm ultracentrifuge tube (Ultraclear, Beckman, Palo Alto, CA) from the bottom to the top, with 1.5 ml of sucrose at a density of 1.30 g/ml, 3 ml at 1.20 g/ml, 4.5 ml at 1.10 g/ml, and 3 ml at 1.006 g/ml medium. The sample in the sucrose gradient was then centrifuged in an SW41-Ti swing rotor (Beckman, Palo Alto, CA) at 16°C for 48 h at 160,000 × gav. Following density gradient centrifugation, twelve 1.0-ml fractions were collected with a mircopipette from the top gradient. The final fraction was stirred to resuspend the pellet. The density of each fraction was determined using a density meter, DMA35N (Anton Paar, Graz, Austria).

### Preparation of lipid emulsions (EM) and apoE-EM complex

Egg yolk phosphatidylcholine (PC) was kindly provided by Asahi Kasei (Tokyo, Japan). Triolein (TO) was purchased from Sigma (St. Louis, MO). Lipid emulsions were prepared by the method described previously [36] using a high-pressure emulsifier (Nanomizer System YSNM-2000AR; Yoshida Kikai Co., Nagoya, Japan). The mixture of TO and PC at a weight ratio of 1:1 was suspended in 50% glycerol in 10 mM Tris-HCl buffer (pH 7.4) containing 150 mM NaCl, 1 mM EDTA and 0.01% NaN_3_, and subsequently emulsified under 140 MPa of pressure at 60°C. Glycerol was exhaustively removed by dialysis against PBS overnight. The contaminating vesicles and larger particles were removed by ultracentrifugation. The weight-averaged particle size of emulsions was 34.7 ± 5.2 nm determined from dynamic light scattering measurements (Photal LPA-3000/3100; Otsuka Electronic Co., Osaka, Japan). The concentrations of TO and PC were determined using enzymatic assay kits purchased from Wako Pure Chemicals (Osaka, Japan). After ultracentrifugation, the weight ratio of TO/PC was 1.63 ± 0.07 (mean ± S.D., n = 5). For the generation of the apoE-EM complex, apoE at various concentrations, from 0.001 to 30 μg/ml, was incubated with EM at a PC concentration of 50 μg/ml in a 5 ml solution for 1 h at room temperature. The apoE-EM complex was isolated and assayed by the ultracentrifugation method as previously reported [24,37].

### Lipid analysis

The extraction of lipids and the subsequent determination of the amounts of cholesterol and phospholipids in the HDL fraction were carried out according to previously described methods [9]. Aliquots (1.0 ml) of conditioned culture media were transferred to clean glass tubes containing 5.0 ml of chloroform: methanol (2:1 v/v). The organic phases were removed, evaporated under N_2 _gas, followed by redissolution in 50 μl of isoprapanol for lipid assay. The amount of total cholesterol was determined using a cholesterol determination kit, LTCII (Kyowa Medex, Tokyo, Japan). The amount of phospholipids was determined using a phospholipid determination kit, PLB (Wako, Osaka, Japan). For the determination of PC level in the apoE+EM complex in the presence of 5% β-mercaptoethanol, PC was extracted with chloroform: methanol (2:1 v/v) as described above. The organic phases were removed, evaporated under N_2 _gas. The samples were redissolution in 20 μl of chloroform: methanol (2:1 v/v) and 5 μl of each solution was spotted on a chromarod-SIII quartz rod and analyzed by thin-layer chromatography/flameionization detector (Iatroscan MK-5 ; Iatron Lab., Inc., Tokyo, Japan).

### Determination of amount of cholesterol and phosphatidylcholine released from neurons labeled with [^14^C]acetate

Neurons cultured for 2 days were labeled with 37 Bq/ml [^14^C] acetate (DuPont NEN) for another 2 days. Two days later, these neurons were washed three times with 1.5 ml of DMEM and incubated in DMEM containing reagents such as apoE3-HDL, apoE4-HDL, apoE3-EM, or apoE4-EM at various concentrations. Aliquots of 1.0 ml each of the conditioned culture media were transferred to clean glass tubes containing 5.0 ml of hexane:isopropanol (3:2 v/v). For the extraction of intracellular lipids, dried cells were incubated in hexane:isopropanol (3:2 v/v) for 1 h at room temperature. The solvent from each sample was evaporated and the organic phases were redissolved in 20 μl (for the condition medium) and 200 μl (for the cells) of chloroform, and 10 μl of each sample was spotted on activated silica gel high-performance thin-layer chromatography (HPTLC) plates (Merck, Darmstadt, Germany); the lipids were separated by sequential one-dimensional chromatography using chloroform: methanol: acetic acid: water (25:15: 4: 2, v/v/v/v), followed by another run in hexane: diethylether: acetic acid (80: 30: 1). [^14^C]-Cholesterol and [^14^C]-phosphatidylcholine were used as standards. The chromatography plates were exposed to radiosensitive films and each lipid was visualized and quantified with BAS2500 (Fuji Film, Tokyo, Japan).

### Immunoblot analysis

Samples of each fraction were dissolved in the sample buffer consisting of 100 mM Tris-HCl (pH 7.4), 10% glycerol, 4% SDS, 10% mercaptoethanol and 0.01% bromophenol blue, and analyzed by 4–20% gradient Trids/tricine SDS-PAGE as previously reported [38]. The separated proteins were transferred onto Immobilon membranes with a semidry electrophoretic transfer apparatus (Nihon Eido, Tokyo, Japan) using a transfer buffer (0.1 M Tris, 0.192 M glycine and 20% methanol). Blots were probed for overnight at 4°C with a goat anti-apoE polyclonal antibody, AB947 (1: 2,000; Chemicon, Temecula, CA). Bands were detected using an ECL kit (Amersham Pharmacia Biotech, UK). For the determination of the concentration of apoE released into the culture medium, signals corresponding to apoE of each sample in the immunoblot membrane were quantified by densitometry using NIH image software, at varying concentrations of synthetic apoE protein (Wako, Tokyo, Japan) as standards. Standard signals were demonstrated to be linear in the range of apoE protein amounts from 0 to 2 μg per lane. ApoE concentrations in the HDL fraction and apoE-EM complex fraction within this range were used for analysis. For immunoblot analysis using anti-ABCAl antibody, the cultured neurons in a 6-well plate were washed in cold PBS and harvested in 500 μl of 50 mM Tric-HCl (Ph 7.4) solution containing 2 mM EGTA. The cell lysates were sonicated and centrifuged at 700 rpm for 10 min at 4°C. The supernatant of each sample was centrifuged at 14,000 rpm for 20 min at 4°C, and the pellet fractions were resuspended in DW containing 0.45 M Urea, 0.1% TritonX-100, and 0.05% Dithiothretol and used for immunoblot analysis as described above.

### Statistical analysis

StatView computer software (Windows) was used for statistical analysis. Statistical significance of differences between samples was evaluated by multiple pairwise comparison among the sets of data using ANOVA and the Bonferoni t-test.

## Competing interests

The author(s) declare that they have no competing interests.

## Authors' contributions

JSG carried out major part of the experiments. SM and TH prepared microemulsion. MK and SCF generated and provided ApoE3- and ApoE4-knock-in mice. KY contributed to interpret the results and make critical intellectual comments. MM participated in its design and coordination and was involved in the interpretation of the results and in drafting the manuscript.
